# Familial Syphilis

**Published:** 1916-04

**Authors:** 


**Familial Syphilis.** Jeans, Am. Jour. Dis. of Chn., Jan. has investigated 100 families. Hereditary germ transmission of syphilis has not been demonstrated in any instance and seems unlikely. It is probable that all mothers of hereditary syphilitics are themselves syphilitic and this was demonstrated by the Wassermann test in 85% of a series of 86. The 100 families gave a total of 331 pregnancies. 30% of the pregnancies resulted in abortion, 9% in still births. Of the 61% of living births (202 children born alive) 24% had died. Of the 154 children surviving, 80% were syphilitic. This leaves almost exactly 10% of the total pregnancies as yielding viable, healthy offspring. Kassowitz's rule of decreasing virulence of infection with passage of time, was noted.

Vander Bogert, ibid. reports a case of twins, one syphilitic and Mongolian in appearance like an elder brother, the other twin being healthy.

